# Handheld Cellphone Use and Risky Driving in Adolescents

**DOI:** 10.1001/jamanetworkopen.2024.39328

**Published:** 2024-10-17

**Authors:** Catherine C. McDonald, Kevin Rix, Jeffrey P. Ebert, Subhash Aryal, Ruiying Xiong, Douglas J. Wiebe, M. Kit Delgado

**Affiliations:** 1 Department of Family and Community Health, University of Pennsylvania School of Nursing, Philadelphia; 2 Penn Injury Science Center, University of Pennsylvania, Philadelphia; 3 Department of Pediatrics, University of Pennsylvania Perelman School of Medicine, Philadelphia; 4 Center for Injury Research and Prevention at the Children’s Hospital of Philadelphia, Philadelphia; 5 Department of Health Promotion and Behavioral Science, University of Texas Health, Houston; 6 Penn Medicine Nudge Unit, University of Pennsylvania Perelman School of Medicine, Philadelphia; 7 Department of Emergency Medicine, University of Pennsylvania Perelman School of Medicine, Philadelphia; 8 Department of Nursing Faculty, Johns Hopkins School of Nursing, Baltimore, Maryland; 9 Department of Medicine, University of Pennsylvania Perelman School of Medicine, Philadelphia; 10 University of Michigan, Ann Arbor

## Abstract

**Importance:**

Handheld cellphone use while driving is associated with increased motor vehicle crash risk among adolescents.

**Objective:**

To examine the association of handheld cellphone use while driving with kinematic risky driving (KRD) events—hard braking and rapid acceleration—in adolescent drivers.

**Design, Setting, Participants:**

Adolescents aged 16.50 to 17.99 years licensed 365 days or less in Pennsylvania were eligible to participate in this cross-sectional study. Enrollment occurred from July 29, 2021, to June 6, 2022. Participants downloaded a smartphone telematics cellphone app to record driving data for 60 days.

**Exposures:**

Trip characteristics, including frequency, length, and duration; presence of speeding; frequency and duration of handheld cellphone use; time of day; and presence of precipitation.

**Main Outcomes and Measures:**

Kinematic risky driving events per 100 miles driven. Zero-inflated Poisson regression models examined whether individual characteristics and trip characteristics were associated with KRD. Incidence rate ratios were computed.

**Results:**

Of 405 adolescents who responded to recruitment, 151 enrolled, 140 completed study procedures, and 119 with 12 360 trips were included in the analytic sample (60 female participants [50.4%]; mean [SD] age, 17.2 [0.4] years). Adolescents drove a mean (SD) of 103.8 (65.7) trips, 565.0 (487.3) miles, and 25.1 (19.3) hours. Adolescents had minimal night trips (1.5% [192]), and few trips with precipitation present (9.0% [1097]). Speeding occurred in 43.9% (5428) of the trips and handheld cellphone use occurred in 34.1% (4214) of the trips. Kinematic risky driving events occurred in 10.9% (1358) of the trips at a rate of 2.65 per 100 miles. In adjusted models, increased KRD events were associated with handheld cellphone use (incidence rate ratio [IRR], 2.62; 95% CI, 1.53-4.48), speeding (IRR, 2.12; 95% CI, 1.06-4.26), and minutes driving (IRR, 1.02; 95% CI, 1.01-1.02). Trips at night, precipitation presence, licensure for less than 6 months, and sex were not associated with increased KRD events.

**Conclusions:**

In this cross-sectional study of adolescent drivers, trips with handheld cellphone use and speeding were associated with higher rates of KRD, while individual characteristics were not. The findings suggest that smartphone telematics apps provide an opportunity to observe behaviors as well as surveil changes due to intervention efforts.

## Key Points

**Question:**

Is handheld cellphone use associated with kinematic risky driving events (hard braking and acceleration) in newly licensed adolescent drivers?

**Findings:**

In this cross-sectional study analyzing 119 adolescents, a smartphone telematics app measuring modifiable risky driving behaviors detected handheld cellphone use in 34.1% of trips. Handheld cellphone use and speeding were associated with an increased risk of kinematic risky driving events.

**Meaning:**

The findings of this study suggest that smartphone telematics apps provide an opportunity to surveil modifiable driving behaviors in adolescents.

## Teaser

This cross-sectional study examines kinematic risky driving events (hard braking and rapid acceleration) associated with handheld cellphone use among adolescents while driving.

## Introduction

Motor vehicle crashes (MVCs) are a leading cause of adolescent death and disability.^1,2,3^ In addition, in 2021, 5565 people of all ages died in MVCs involving drivers aged 15 to 20 years.^4^ Driver distraction is a major contributor to adolescent driver MVCs^5,6^ and includes visual, physical, and cognitive components (eg, not looking at the roadway ahead, hands not on the wheel, and mind off the task of driving).^7^ Adolescents are particularly susceptible to the MVC risks associated with distracted driving, even in the first months of licensure. Among these distraction-related MVC risks is handheld cellphone use while driving.^8,9,10^

Current prevention efforts fall short in addressing cellphone-related MVCs, events that are preventable. Policies restricting cellphone use while driving have shown mixed effectiveness, with limited long-term benefits.^11^ However, primary enforced distracted driving laws show promise.^12^ Blocking technologies that disable mobile devices are available to intervene at the individual level,^13^ although assessing outcomes can be difficult and uptake is limited by acceptability. The data available on cellphone use while driving is a limiting factor in characterizing the extent and nature of handheld cellphone use while driving, as well as intervention outcomes.

In studies with either large samples or population-based samples, adolescents self-report cellphone use while driving.^14,15,16^ For example, the most recent (2019) data from the nationally representative Centers for Disease Control and Prevention Youth Risk Behavior Survey found that 39% of adolescents report texting or emailing while driving in the prior 30 days.^16^ Such measures can become quickly outdated or limited in the mode of assessing adolescent cellphone communication (eg, asking about texting when adolescents use apps to communicate), driving scenario (car stopped, traveling at low speeds or high speeds), and meaningful differences between response options (the intervals of days vs trips).^17^ In-vehicle monitoring data of adolescents in Virginia (data collected between 2006 and 2008) observed that cellphone use while driving increased from 8% of drive time at 3 months of licensure to 11% at 12 months, indicating increasing use as adolescents gain more driving experience.^8^ Data collections such as these are important; however, reliance on in-vehicle monitoring using cameras with randomly sampled periods and high-risk driving events is labor- and time-intensive and limits the understanding of handheld cellphone use while driving. Likewise, studies that rely on roadway observations may be biased in their assessments of age, sex, gender, and other individual-level driver characteristics.^10^ Results from this variety of methods have shown mixed results on the differences between males and females related to their cellphone use while driving^16,18^ or have not reported sex-associated differences, likely due to small sample size.

Kinematic risky driving (KRD) events have been identified as driving behavior metrics associated with elevated crash risk.^19,20^ Kinematic risky driving events can include elevated gravitational (*g*)-force events, such as hard braking and rapid acceleration, and have often been the target of adolescent driving interventions to improve safe driving.^21,22^ Cellphone use, in particular handheld cellphone use, is associated with increased MVC risk.^8^ Given the increase of cellphone use in general among adolescents, as well as the consistent expansion of cellphone use while driving, it is important to examine whether there is an association of cellphone use with KRD events to better target intervention efforts.

With limitations in current data collection methods, it can be difficult to accurately surveil changes, implement interventions based on data, or measure the outcomes of interventions. Smartphone telematics apps, which use GPS, accelerometers, and other sensors built into existing cellphone hardware to measure driving behavior and cellphone use, are another method. They have transformative potential for scalable interventions in the ability to assess and deliver automated smartphone-enabled behavioral interventions that involve feedback, notifications, and/or education.^23,24,25,26^ The objective of this study was to examine whether there was an association of handheld cellphone use while driving with KRD events in adolescent drivers.

## Methods

Pennsylvania adolescents licensed for 365 days or less were prospectively enrolled between July 29, 2021, and June 6, 2022, during this cross-sectional study. On-road driving data were collected through a smartphone telematics app (Way to Drive, a white-label telematics app developed at the University of Pennsylvania and produced by Cambridge Mobile Telematics) that participants downloaded at enrollment. Data were collected for approximately 60 days. Surveys were completed via the Research Electronic Data Capture (REDCap) platform hosted at the University of Pennsylvania. Parents provided written informed consent and participants provided written informed assent. Participants were compensated $85 for completing the study procedures. The protocol was approved by the institutional review board of the University of Pennsylvania and a cooperative agreement with the Children’s Hospital of Philadelphia. This study followed the Strengthening the Reporting of Observational Studies in Epidemiology (STROBE) reporting guideline.

### Sample Selection Criteria

Recruitment occurred through mailings, emails, flyers, databases for clinical trials (eg, ResearchMatch, iConnect), social media postings, and the Recruitment Enhancement Core, using emails and letters distributed to individuals in the Children’s Hospital of Philadelphia electronic health record. Eligible participants were adolescents aged 16.5 to 17.99 years licensed in Pennsylvania for 365 days or less at enrollment, could read and write in English, had access to the internet and a personal email address, owned a smartphone, and had a parent who could give permission for participation. Siblings of enrolled participants were ineligible. Sex stratification for enrollment was applied to achieve equal numbers of male and female participants in the study.

We used PASS, version 16.01 software (NCSS LLC) to determine the sample size and the minimum odds ratios (ORs) that we could estimate based on speeding data in a preliminary study. With a sample of 125 and α = .05 for 2 group comparisons in the initial models (eg, low KRD vs high KRD), we anticipated having greater than 83% power to detect ORs of 1.75 or more for mean trip characteristics with handheld phone use in 40% to 50% of trips or driving time, greater than 84% power to detect ORs of 2.0 or more at 20% to 30%, greater than 85% power to detect ORs of 2.5 or more at 10%, and greater than 80% power to detect ORs of 3.5 at 5% and 5.5 at 2.5%. An adjustment was made to assume an *R*^2^ value of 0.10 between other variables in the model.

### Way to Drive App and Data Reduction

After enrollment and completion of the baseline survey, adolescents installed the Way to Drive app, which monitored driving trips for 60 days. Participant enrollment and data transfer are managed through a secure application programming interface with Way to Health. The app uses the same driver classification and risky behavior detection algorithms (version 2.3.0) as leading usage-based insurance apps. In a validation study, the app’s driver and nondriver classifications were 97% accurate with a sample of adult drivers.^27^

### Trip Information

Information collected by the app about each trip (origin to destination; ie, 1-way) included predicted mode of transportation (eg, car, bus, train), driver trip prediction score (0-100) as to whether the adolescent was a driver on the trip, trip characteristics (date, time, GPS location), mileage, duration, presence and duration of handheld cellphone use, instances of hard-braking events with rapid deceleration (≤−0.40*g*),^28^ instances of hard-acceleration events (≥0.35*g*),^29^ and presence and duration of speeding events (>10 mph over posted road speed limit or >80 mph if no speed limit data available). Total handheld cellphone use, measured in seconds, was the sum of the amount of time spent using a device for a handheld phone call (call handheld) and the time spent using the phone for typing and swiping (noncall handheld) while traveling at more than 8 mph. Handsfree cellphone use was not included in this analysis (eg, using the phone as GPS, music/podcasts, or having a phone call through speakerphone/car audio). Additional Pennsylvania county-level weather data (98.5% of trips) were collected from the National Oceanic and Atmospheric Administration (NOAA) to include precipitation data queried from the NOAA Climate Data Online search tool.^30^ Nighttime driving was characterized by trips occurring between 11 pm and 5 am, consistent with Pennsylvania Graduated Driver Licensure provisions.^31^ For analytic purposes, trip characteristics were coded as dichotomous variables for a given trip: nighttime trip (yes/no), precipitation present (yes/no), speeding present (yes/no), and handheld cellphone use present (yes/no).

### KRD Events

The main outcome was KRD events, which were calculated for a trip as a sum of hard braking (≤−0.40*g*) and rapid acceleration (≥0.35*g*) events in a trip.^29,32^ To reduce overcounting in the case of rapid successive braking or accelerator tapping, the first event was retained and subsequent like events in a 3-second window were excluded.

### Demographic Data

Participants completed baseline questionnaires, self-reporting demographic information, including race and ethnicity, and driving characteristics via REDCap. For race, reporting options included Asian, Black or African American, American Indian/Alaska Native, Native Hawaiian or Other Pacific Islander, White, and Other. Participants were asked to check all that apply. For ethnicity, participants were asked “Would you describe yourself as either Hispanic or Latino?” and could answer yes, no, or unsure. Race and ethnicity were ascertained to provide an overall description of the sample. Participants also completed short survey check-ins at weeks 2 and 6 and full questionnaires at weeks 4 and 8 over the course of 8 weeks of enrollment in the study.

### Statistical Analysis

Data analysis was conducted using Stata, version 17.0 (Stata Corp LLC) and SAS, version 9.4 (SAS Institute Inc); geographic information system mapping was completed using ArcMap, version 10.7.1 (Esri). Way to Drive JavaScript Object Notation trip files were first processed using an R script, version 4.2.3 (R Foundation for Statistical Computing) that summarized key driving metrics and the algorithm’s driver and nondriver classification. Trip data were reduced to an analytic dataset. Duplicate, nondriver, and noncar trips were removed. To conservatively sample trips where there was a high degree of confidence that the participant was driving, only trips with 90% or more driver prediction scores were included in the analytic sample. Trips with 0 valid miles (trips in which the app could not detect KRD events due to missing sensor data) and trips with 0 valid usage time (trips in which the vehicle never exceeded 8 mph or in which the app was unable to detect phone use or nonuse) were also removed. Only drivers who recorded at least 25 trips were included. Drivers with less than that amount were excluded from analysis because of concern that their small number of trips would not accurately represent their driving behaviors. Those excluded for less than 25 trips had a mean (SD) of 11.0 (6.9) trips over their entire 60-day monitoring period.

We calculated means (SDs) to describe continuous outcomes. Frequency distribution was obtained for categorical data. We used graphic approaches, including histogram and boxplots, to evaluate normal distribution assumptions. To compare study participants’ driving and trip characteristics, we used a 2-sample *t* test for normally distributed variables and nonparametric Wilcoxon rank sum test for nonnormal data. Categorical characteristics were compared using the χ^2^ test and Fisher exact test.

Our primary outcome variable was the number of KRD events for each trip, and we considered the following predictor variables for the trip: any handheld cellphone use (yes/no), night trip (11 pm-5 am) (yes/no), precipitation present (yes/no), sex (male/female), trip duration (minutes), licensed 6 months or more (yes/no), and speeding present (yes/no).

We fit a 0-inflated Poisson (ZIP) regression model under the generalized estimating equation framework to evaluate whether there was an association between number of KRD events and the predictor variables. A ZIP regression model was used to account for excess 0 KRD events observed in the data, while the generalized estimating equation approach was used to account for nesting of trips within each participant. We used sex, speeding presence, and handheld cellphone use presence as predictors in the logistic portion of the ZIP model. The count regression component used all of the predictors listed above. Valid miles per 100 miles driven was used as an offset in our ZIP model. To assess the robustness of our findings against unmeasured and residual confounding, we calculated the *E* value for variables significant in the adjusted analysis.^33^ Incidence rate ratio (IRR) and corresponding 95% CI was calculated for predictors in the Poisson regression model portion, and a threshold of *P* < .05 was considered statistically significant.

## Results

Of the 405 individuals who responded to recruitment, 151 adolescents were enrolled, 140 completed study procedures, and 119 (female, 60 [50.4%]; male, 59 [49.6%]) were included in the analytic sample. Mean (SD) age was 17.2 (0.4) years. The Figure outlines the inclusion of the analytic sample of adolescents and trips. Table 1 presents the demographic characteristics. Participants reported the following racial groups: Asian, 3 (2.5%); Black or African American, 4 (3.4%); White, 103 (86.6%); Multiple, 6 (5%); and Other 3 (2.5%). Participants reported the following for ethnicity: non-Hispanic, 100 (84.0%), and Hispanic, 11 (9.2%); 8 (6.7%) were not reported. We identified no significant difference across predictor variables between male and female participants.

**Figure.**
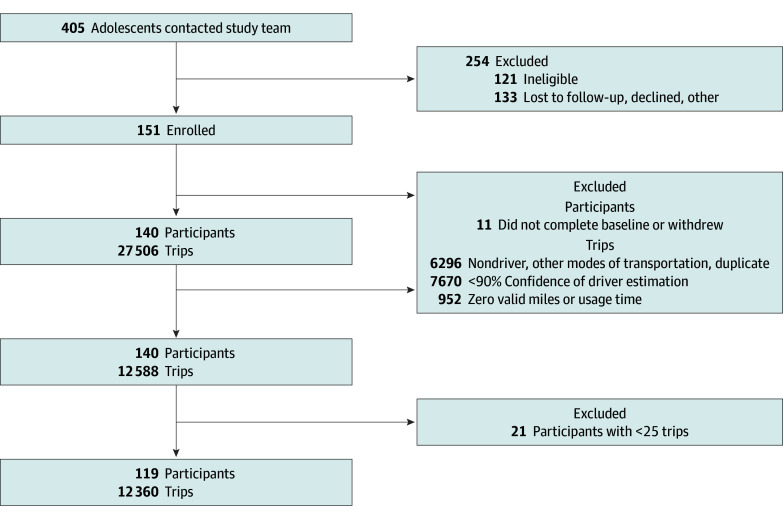
Flow Diagram of Participant Inclusion

**Table 1.** Teen Driver Self-Reported Individual Characteristics

Characteristic	Participants, No. (%)			*P* value
	Overall sample (N = 119)	Males (n = 59)	Females (n = 60)	
Age, mean (SD), y	17.2 (0.4)	17.2 (0.4)	17.1 (0.3)	.07
Length of licensure, mean (SD), mo	5.4 (3.2)	5.6 (3.4)	5.1 (3.0)	.15
Cellphone type (% iPhone)	91.6 (109)	89.8 (53)	93.3 (56)	.75
Past month days driven, mean (SD)	22.4 (7.5)	21.8 (8.4)	23.0 (6.6)	.31
Past month, % had >1 other teen in car while driving	64 (54.2)	30 (50.9)	34 (57.6)	.09
Past month, % read text while driving >6 d	32 (27.1)	17 (28.8)	15 (25.4)	.08
Past month, % wrote text while driving >6 d	28 (23.7)	14 (23.7)	14 (23.7)	.99
Past month, % used social media while driving >6 d	7 (5.9)	5 (8.5)	2 (3.3)	.27

Table 2 outlines the driving and trip characteristics. There was a total of 12 360 trips (males, 5928; females, 6432), for 67 245 miles (males, 31 972; females, 25 273) and 2070 hours (males, 992; females 1078). Adolescents drove a mean (SD) of 103.8 (65.7) trips, 565.0 (487.3) miles, and 25.1 (19.3) hours. Trips were relatively short in distance (mean [SD], 5.3 [2.8] miles) and duration (mean [SD], 14.3 [6.0] minutes). There was little nighttime driving among the sample (1.5% of all trips [192]), and 9.0% (1097) of trips had precipitation present. Speeding was present in 43.9% (5428) of the trips, although the duration per hour of driving was low at 2.0 minutes per hour. Handheld cellphone use was detected in 34.1% (4214) of the trips, with mean (SD) duration of cellphone use during these trips of 2.1 (4.0) minutes per trip. Kinematic risky driving events occurred in 10.9% (1358) of the trips at a rate of 2.65 per 100 miles. In bivariate comparisons, males were significantly more likely to have a trip with speeding occurring, drive with any precipitation, a trip with a KRD event, and a KRD event per 100 miles driven.

**Table 2.** Teen Driver Trip Characteristics

Characteristic	Mean (SD)			*P* value
	Overall sample (N = 119)	Males (n = 59)	Females (n = 60)	
Trip frequency	103.8 (65.7)	100.4 (64.2)	107.2 (67.6)	.58
Total distance, miles	565.0 (487.3)	541.8 (508.7)	587.8 (468.5)	.61
Trip duration, min/trip	14.3 (6.0)	14.0 (6)	14.6 (6.0)	.61
Miles/trip	5.3 (2.8)	5.2 (2.6)	5.3 (3.0)	.73
Nighttime driving duration, total min	21.8 (46.3)	28.7 (56.7)	15.0 (32.1)	.11
Speeding duration, min/h	2.0 (2.2)	2.2 (2.2)	1.8 (2.2)	.32
Handheld use duration, min/trip	0.68 (1.04)	0.70 (1.1)	0.67 (.94)	.86
Handheld use duration among trips with use, min/trip	2.1 (4.0)	2.17 (4.1)	2.01 (4.1)	.19
KRD frequency, events/100 miles	2.65 (2.5)	3.33 (3.1)	1.9 (1.4)	<.001
Trips with night driving, No. (%)	192 (1.5)	129 (2.1)	63 (0.98)	.55
Trips with precipitation, No. (%)	1097 (9.0)	555 (9.6)	542 (8.5)	.01
Trips with speeding, No. (%)	5428 (43.9)	2829 (47.7)	2599 (40.4)	<.001
Trips with handheld cellphone use, No. (%)	4214 (34.1)	2307 (38.9)	1907 (29.7)	<.001
Trips with KRD events, No. (%)	1358 (10.9)	779 (13.1)	579 (9.0)	<.001

Abbreviation: KRD, kinematic risky driving.

In the adjusted model, handheld cellphone use (IRR, 2.62; 95% CI, 1.53-4.48; *P* < .001), speeding (IRR, 2.12; 95% CI, 1.06-4.26; *P* < .001), and minutes driving (IRR, 1.02; 95% CI, 1.01-1.02; *P* < .001) were associated with KRD events. All other variables were statistically nonsignificant (Table 3).

**Table 3.** Association of Driver and Trip Characteristics With Kinematic Risky Driving Events^a^

Characteristic	Adjusted IRR (95% CI)	*P* value	E value for IRR	Lower bound of 95% CI for E value
Handheld cellphone use, yes/no	2.62 (1.53-4.48)	<.001	4.68	2.44
Speeding present, yes/no	2.12 (1.06-4.26)	<.001	3.66	1.31
Trip duration (minutes)	1.02 (1.01-1.02)	<.001	1.15	1.12
Night trip, yes/no	0.87 (0.51-1.51)	.62	NA	NA
Precipitation present, yes/no	0.96 (0.80-1.14)	.65	NA	NA
Sex (male)	1.46 (0.83-2.68)	.21	NA	NA
Licensed ≥6 mo, yes/no	0.97 (0.67-1.41)	.87	NA	NA

Abbreviations: IRR, incidence rate ratio; NA, not applicable.

^a^ Total of 12 178 with Pennsylvania weather available.

The point estimate of the *E* value for handheld cellphone use was 4.68, with a lower bound of 95% CI of 2.44, which indicates that the observed IRR of 2.62 would only be explained by an unmeasured confounder of magnitude greater than 4.6 times above what was explained by the measured confounder adjusting for other variables in the model. Similarly, an unmeasured confounder with an IRR of 3.66 above that explained by the observed confounders would be needed to explain the observed IRR of 2.12 between speeding and KRD events, and an unmeasured confounder of IRR of 1.15 above that related to trip duration and KRD events.

## Discussion

Among adolescent drivers licensed for less than 1 year, a smartphone telematics app indicated a notable amount of speeding, handheld cellphone use while driving, and KRD events. Among these drivers, there was little nighttime driving and infrequent driving with precipitation present. Overall, individual characteristics were not associated with KRD events in this sample; rather, it was driving behaviors, such as speeding and handheld cellphone use. Incorporating weather data helped identify the conditions in which adolescents drove and revealed that precipitation was not associated with KRD events.

Smartphone telematics app data are a unique source of information about contributors to risky driving events, as well as overall driving behavior. The breadth and depth of the data in this study with more than 12 000 trips allowed for an accounting of trip characteristics such as time of day, weather, and risky driving events. In this study, the rate of KRD events was in the range of what has been reported by other adolescent driving studies.^19,20,21^ The behaviors associated with increased KRD events are modifiable—handheld cellphone use and speeding are driving behaviors that intervention efforts can address for adolescent drivers. In 2022, speeding was a factor in 30% of adolescent (age, 15-18 years) driver fatal MVCs,^34^ and 6% of drivers aged 15 to 20 years involved in fatal MVCs were distracted.^5^ These 2 key behaviors identified in smartphone telematics applications can be targeted for intervention.

Male adolescents are at a higher risk for MVCs and thus are thought to be riskier drivers,^35^ perhaps due to sex differences in the developing adolescent brain.^36^ In unadjusted results, we saw that male sex was associated with a higher IRR of KRD events. In adjusted results, the IRR decreased to nonsignificance. It is possible that in a larger sample, this finding may have been significant. Regardless, the decrease in significance may be interpreted as evidence that the association between sex and KRD is at least partially mediated by speeding and handheld cellphone use. Earlier research has reported that one reason males have more KRD events is that they are more likely to speed.^37^ Our study adds new information, suggesting that sex differences in cellphone use may also contribute to differences in KRD events.

Cellphone use is ubiquitous among adolescents, and our data suggest that handheld cellphone use while driving occurs in a substantial proportion of trips. Not every trip had handheld cellphone use while driving; however, when it occurred, it was for a mean of 2.1 minutes per trip. Given the mean trip length was less than 15 minutes, this difference is substantial. Further investigation of adolescent drivers who have different driving norms due to geographic location (ie, rural vs suburban vs urban) outside of Pennsylvania is needed. There may be variations among adolescents from rural areas who drive further distances for longer durations, as well as those from urban areas with different roadway characteristics of lower speeds.^38^

Smartphone telematics offer a powerful tool for adolescent driver safety interventions.^26,39,40,41^ Using a smartphone telematics app to detect both cellphone use and KRD events while driving without the need for in-car monitoring or direct observation provides a broader opportunity to reach large-scale populations of adolescent drivers who may be hesitant for in-vehicle video monitoring. In addition, a smartphone app does not contain the bias often typically associated with self-report. Usage-based insurance programs already deploy smartphone telematics to assess driver risk,^42^ setting an example for how such interventions may be successful for adolescent drivers.

### Limitations

This study has limitations. Ours was a convenience sample of participants in the greater Philadelphia area who held a Pennsylvania license, and only those who expressed interest in participating were enrolled. As such, findings may not generalize to all newly licensed adolescent drivers. However, the sample of 140 enrolled and 119 drivers analyzed during 60 days provided thousands of miles driven and thousands of unique drives that reflected a range of driving behaviors and conditions experienced by newly licensed adolescent drivers. In addition, by aiming for equal distribution of male and female drivers, we were able to examine sex-specific differences among newly licensed adolescent drivers in our population. We were not able to estimate sequence or causality with these analyses. Although this is a limitation, these results still provide important information on characterizing cellphone use while driving, speeding, and KRD events in a scalable approach.

The smartphone app measuring driving behaviors is also imperfect. Trips are classified as driver or nondriver (ie, passenger) via an algorithm that has been found to be 97% accurate with a sample of adult drivers.^27^ The algorithm is biased to predict driver trips and therefore may be less accurate for newly licensed adolescent drivers, who are more likely to be passengers. We tried to minimize the risk of including nondriver trips by only analyzing trips in which the algorithm was at least 90% confident vs a default threshold of 50% confidence that the adolescent was the driver.

## Conclusions

In this cross-sectional study of adolescent drivers licensed for 365 days or less, trips with handheld cellphone use and speeding were associated with higher rates of KRD, while individual characteristics, such as length of licensure and sex, and environmental factors, such as driving at night and during precipitation, were not. This study suggests how a novel approach can provide insight into normally difficult-to-measure adolescent driving behaviors. Using a smartphone telematics app to remotely collect epidemiologic data creates opportunities for testing behavioral interventions in a nonresearch setting. This study identified an increased incidence of KRD associated with handheld cellphone use and speeding. Further examination of the situations in which this cellphone use and speeding occurred could inform safe driving interventions tailored to this population. This study is an important step toward reducing MVCs among adolescents.

## Notes

Supplement.
Data Sharing Statement
