# Supplementary material for: Handheld Cellphone Use and Risky Driving in Adolescents
Source: JAMA Netw Open. 2024 Oct 17;7(10):e2439328. doi: 10.1001/jamanetworkopen.2024.39328 (PMC11581603; doi:10.1001/jamanetworkopen.2024.39328)
Supplement: Supplement. — Data Sharing Statement [file jamanetwopen-e2439328-s001.pdf]

# Data Sharing Statement

McDonald. Handheld Cellphone Use and Risky Driving in Adolescents. *JAMA Netw Open*. Published October 17, 2024. doi:10.1001/jamanetworkopen.2024.39328

**Data**
**Data available:** Yes
**Data types:** Deidentified participant data
**How to access data:** Deidentified data upon request pursuant to regulations.
**When available:** With publication

**Supporting Documents**
**Document types:** None

**Additional Information**
**Who can access the data:** Qualified researchers
**Types of analyses:** Re-analyses
**Mechanisms of data availability:** signed DUA
